# Médiastinite compliquant une cellulite cervicale à porte d′entrée dentaire: à propos d′un cas et revue de la littérature

**Published:** 2011-03-15

**Authors:** Issam Serghini, Younés Aissaoui, Youssef Quamouss, Rachid Sedikki, Karim Filali, Mohamed Zoubir, Mohamed Boughanem

**Affiliations:** 1 Service d Anesthésie réanimation, hôpital militaire Avicenne, Marrakech, Maroc

**Keywords:** Cellulite cervicale, infection dentaire, infections à germes anaérobies, retard diagnostic, médiastinite

## Abstract

Les cellulites cervicales ou fasciites cervicales nécrosantes sont des infections des parties molles développées à partir de foyer dentaire ou pharyngé dont le risque, si elles ne sont pas reconnues précocement, est l’extension vers le médiastin. Les premiers signes cliniques sont parfois frustres et peuvent conduire à un retard diagnostique. L’examen clé est la tomodensitométrie cervicale et thoracique. Le traitement consiste en des excisions tissulaires larges et répétées associées une antibiothérapie dirigée contre les germes aréo- et anaérobies. Nous rapportons le cas d’un homme connu diabétique présentant une cellulite cervicale d’origine dentaire compliquée de médiastinite.

## Introduction

Les cellulites cervicales ou fasciites nécrosantes cervicales sont heureusement peu fréquentes. Il s’agit de processus infectieux qui se développent dans des espaces anatomiques cloisonnés. Le risque initial est le retentissement sur les voies aériennes supérieures. Le risque secondaire est l’extension de l’infection au médiastin par diffusion en suivant le trajet des gaines du cou. Ces infections graves doivent être traitées le plus rapidement possible car elles constituent une menace vitale et peuvent laisser des séquelles délabrantes [1].

A travers un cas clinique et une revue de la littérature nous discuterons la prise en charge d’une médiastinite compliquant une cellulite cervicale à point de départ dentaire chez un sujet immunodéprimé.

## Patient et observation

**Figure 1:**
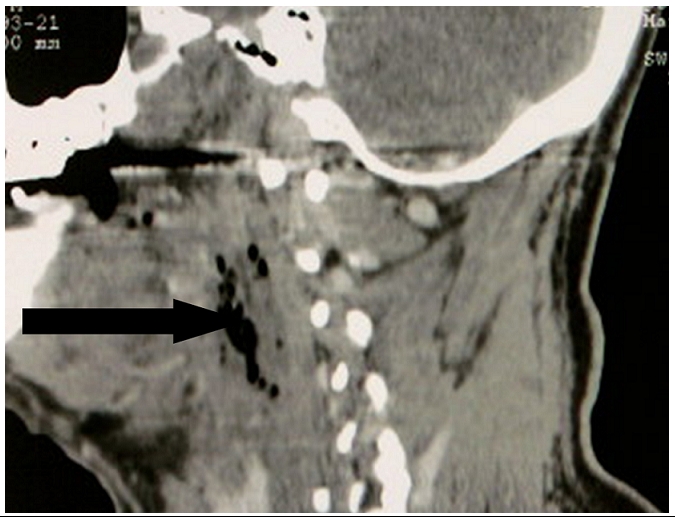
TDM cervicale montrant un phlegmon avec cellulite profonde des espaces cervicaux antérolatéraux chez un patient marocain de 55 ans, diabétiques, avec médiastinite compliquant une cellulite cervicale à porte d’entrée dentaire

**Figure 3:**
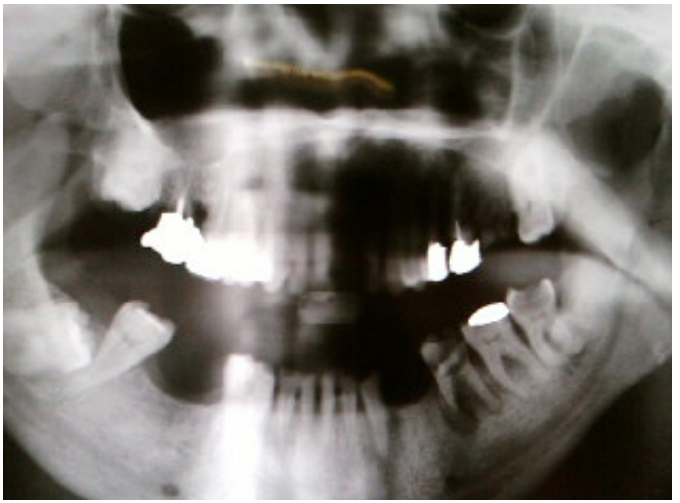
Le panoramique dentaire objective un délabrement de la 3 éme molaire chez un patient marocain de 55 ans, diabétiques, avec médiastinite compliquant une cellulite cervicale à porte d’entrée dentaire

Nous rapportons l’observation d’un homme de 55 ans enseignant de fonction ayant comme antécédents pathologiques un diabète noninsulinodépendant découvert depuis deux mois et des soins dentaires itératifs, qui consulte aux urgences pour une masse latéro-cervicale gauche ferme à la palpation évoluant dans un contexte fébrile et une altération de l’état général. Lexamen clinique à l’admission trouve un patient asthénique, un érythème discret sur la face latéral du cou, une fréquence cardiaque à 87 batt/min et une PA à 110/70 mmHg. L’état respiratoire était normal: fréquence respiratoire 22 cycles/mn, une SaO2 à 96 % à l’air ambiant, une fièvre à 39°. Le bilan biologique initiale montre une hyperleucocytose à 85OOO, une CRP à 350 mg/l et une glycémie à 12 mmol/l. La TDM cervicothoracique révèle un phlegmon avec cellulite profonde abcédée des espaces cervicaux antérolatéraux gauches fusant le long de l’’espace retropharyngolaryngé étendue au médiastin compliqué d’ une médiastinite associé à un épanchement pleural droit (Figure 1 et 2). Le panoramique dentaire objective un délabrement de la 45 (3éme molaire) avec granulome de la racine dentaire (Figure 3). La ponction sous-cutanée sous anesthésie locale après injection d’un petit volume de sérum physiologique (10 ml) de la collection a ramené du pus. L’examen cytobactériologique montre des leucocytes nombreux avec des bactéries abondantes et polymorphes. L’examen direct coloré GRAM : Flore bactérienne très abondantes et mixte évoquant les anaérobies. Une antibiothérapie initiale, adjuvante, probabiliste a été instaurée par voie intraveineuse le plus vite possible avant le bloc amoxicilline+acide clavulanique (augmentin 2g× 3/j) associé à 5-nitro-imidazolés (flagyl 500 mg× 3/j).

**Figure 4:**
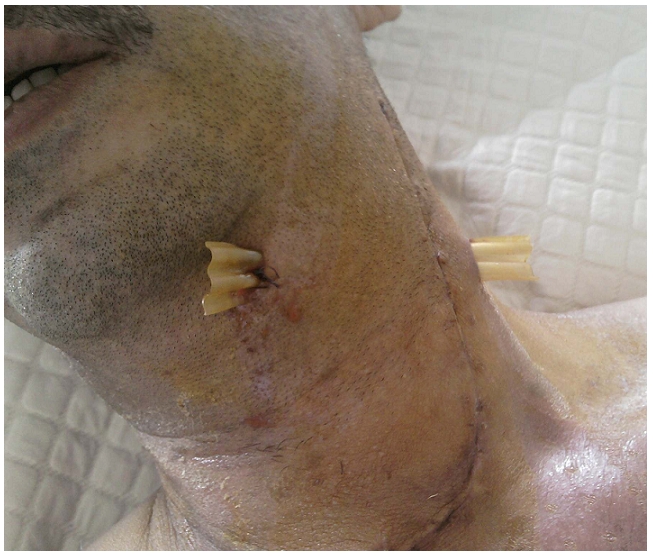
Le foyer infectieux drainé par des lames descendues à partir de la cervicotomie chez un patient marocain de 55 ans, diabétiques, avec médiastinite compliquant une cellulite cervicale à porte d’entrée dentaire

Au bloc opératoire sous anesthésie générale, le foyer infectieux est abordé par une large incision cervicale et le phlegmon abcédé a été drainé par des lames descendues à partir de la cervicotomie (Figure 4). Une thoracotomie a été réalisée pour drainer les collections profondes au niveau du médiastin. Une cellulite au stade collecté justifie d’une prise en charge chirurgicale. Au décours du drainage de la cellulite, l’’extraction de la dent causale a été réalisée. Un traitement complémentaire de la cavité buccale s’est avéré nécessaire pour prévenir tout autre épisode infectieux similaire. La culture retrouve un streptococcus anginosus multisensible. L’antibiothérapie initiale a été par la suite réajustée aux résultats de l’’hémoculture.

## Discussion

Le terme de cellulite cervicale regroupe des entités anatomo-pathologiques variées allant d’une atteinte du derme superficiel jusqu’aux muscles [2]. L’extension de l’infection est très rapide justifiant le caractère urgent d’une prise en charge adaptée. En l’absence d’un traitement chirurgical précoce et bien conduit, linfection va se propager vers le médiastin dans plus de 20 % des cas [3,4]. La médiastinite est l’évolution naturelle d’une cellulite nécrosante cervicale. Il s’agit d’une entité à part entière à différencier des autres types de médiastinites (post-chirurgicales, perforation d’organe creux). La nomenclature anglosaxonne a attribué le nom de descending mediastinitis exclusivement à ce type de médiastinite. L’origine dentaire de la cellulite est identifiée par la plupart des études comme un facteur favorisant l’extension vers le médiastin notamment lorsque les deuxième ou troisième molaires sont infectées. Les facteurs de risque classiques d’immunosuppression comme l’alcoolisme, le diabète, les pathologies néoplasiques, la prise d’anti-inflammatoire non stéroïdiens ou de corticoïdes sont fréquemment incriminés [1]. Les patients sont souvent hospitalisés pour des douleurs persistantes après une extraction dentaire ou par la sensation de masse liée au développement d’un abcès oro-pharyngée, parfois dès le stade de complications pour une dyspnée ou une altération de l’état général. L’examen clinique est souvent initialement pauvre mais peut retrouver un érythème ou un œdème local souvent discret. Le cou devient ensuite rouge tendu et douloureux. La présence de crépitations signe la production de gaz. L’examen doit s’attacher à rechercher des signes d’extension: dysphagie et dyspnée laryngée qui signe un retentissement sur les voies aériennes supérieures. L’état général peut être longtemps conservé et s’altérer brutalement [1].

Le bilan biologique retrouve un syndrome inflammatoire non spécifique. L’essentiel du bilan de localisation et d’extension est fait sur la tomodensitométrie cervicothoracique injectée, réalisée le plus précocement possible. L’injection se fait dans le territoire cave supérieur gauche afin de bien opacifier le tronc brachiocéphalique. Cet examen recherche: le point de départ de l’infection: dentaire ou pharyngée et les signes témoignant de l’atteinte des parties molles: infiltration des tissus, collections, présence de gaz [1].

Dans les cellulites cervicales, la notion déquipe médicochirurgicale est importante dans la prise en charge. Le retard de diagnostic, lattentisme sous antibiothérapie même bien conduite et une chirurgie retardée et/ou insuffisante sont les principaux facteurs expliquant de nombreux échecs.

L’antibiothérapie nest « quadjuvante » dans la stratégie thérapeutique après la chirurgie. Si son intérêt reste limité dans les zones très atteintes par manque de diffusion locale des antibiotiques, elle permet toutefois de limiter lextension de linfection aux zones saines périphériques et sa dissémination hématogène. Comme pour toute infection grave, une dose de charge dantibiotiques est administrée pour favoriser le passage de lantibiotique libre dans les tissus après saturation de protéines transporteuses, puis des doses fortes à intervalles rapprochés, variables selon les antibiotiques. Dans un second temps, lantibiothérapie est adaptée aux résultats des cultures des prélèvements pré et per-opératoires. Elle associe toujours au moins un antibiotique actif contre les bactéries aérobies et un antibiotique actif contre les germes anaérobies. Soixante à quatre-vingtdix pour cent des cellulites cervicales sont polymicrobiennes à flore mixte aéroanaérobie. On retrouve principalement des streptocoques du groupe milleri (anginosus, constellatus, intermedius), des streptocoques pyogènes et quelques staphylocoques dorés ou à coagulase négative et des Prevotella X1. On cible plutôt les streptocoques, ceux du groupe A en particulier, et les anaérobies (souvent sensibles aux bêtalactamines). Lassociation classique comprend pénicilline G à la dose de 30 MU/j (ou amoxicilline : 100 mg/kg par jour) et clindamycine à la dose de 600 mg quatre fois par jour ou rifampicine 10 mg/kg deux fois par jour. On préférera lamoxicilline–acide clavulanique à 2 g/j × 3 associée à la gentamicine haute dose 6–8 mg/kg en une injection quotidienne [5].

En période préopératoire, on peut ponctionner des collections ou des phlyctènes installées ou pratiquer des ponctions sous-cutanées après injection dun petit volume de sérum physiologique (10 ml) au milieu des lésions nécrotiques (sensibilité > 70 %). Les examens directs permettent de débuter une antibiothérapie ciblée qui sera par la suite réajustée aux résultats des cultures. Les hémocultures, bien que systématiques, ne sont positives que dans 10 à 35 % des cas.

Le foyer infectieux est abordé par une large incision cervicale et le médiastin est drainé selon l’extension de l’infection par des lames descendues à partir de la cervicotomie, par sternotomie ou par thoracotomie lorsque les collections sont plus profondes. L’écueil à éviter est un traitement trop conservateur ne permettant pas d’éliminer tous les tissus nécrosés et infectés [6].

En fonction du stade clinique de la cellulite et du degré d’atteinte de la dent causale, ce traitement va de la simple trépanation à l’avulsion de la dent. La trépanation dentaire permet louverture large de la cavité pulpaire et le drainage du foyer apical, préservant la dent; en effet une obturation canalaire pourra être réalisée à distance de cet épisode infectieux. Ce traitement est réservé aux cellulites au stade séreux sans délabrement dentaire trop important, sinon l’avulsion de la dent causale est pratiquée sous anesthésie locale. Une cellulite au stade collecté justifie d’une prise en charge chirurgicale et l’avulsion de la dent causale quelque soit son degré d’atteinte. Le geste est pratiqué au bloc opératoire sous anesthésie générale au décours du drainage de la cellulite. Un traitement complémentaire de la cavité buccale s’avère parfois nécessaire afin de prévenir tout autre épisode infectieux similaire.

La durée de lantibiothérapie varie selon les habitudes des équipes, la gravité de linfection initiale et surtout lévolution du patient. Elle est maintenue à un minimum 15 jours jusquà plusieurs semaines après disparition des signes dinfection locaux et généraux. Certains la préconisent jusquà la fermeture complète des lésions cutanées. La littérature ne donne pas de réponse concrète [5].

L’intérêt de l’oxygénothérapie hyperbare reste controversé. La Haute Autorité de Santé recommande l’oxygénothérapie hyperbare en traitement adjuvant et simplement sur des avis d’experts, en précisant que ces recommandations ne sont pas soutenues par des données de haut niveau de preuve. Son recours ne justifie en aucun cas un transfert médical avant la réalisation d’une chirurgie et la mise en route d’une antibiothérapie adaptée. L’oxygénothérapie hyperbare est aussi utilisée afin d’accélérer la cicatrisation des plaies. Mais, là aussi, les données validant cette indication sont insuffisantes [7].

Il existe peu d’arguments dans la littérature pour recommander un type de pansement particulier, qu’ils soient classiques à base de compresses humides ou plus innovants à base d’alginates de calcium, d’argent ou d’hydrocolloides. Une alternative intéressante aux pansements classiques serait les pansements occlusifs à pression négative type VAC. Ceux-ci permettent une meilleure cicatrisation du site opératoire et une diminution de la charge de travail infirmier en autorisant un changement de pansement toutes les 48 à 72 heures au lieu de plusieurs fois par jour. Lorsqu’ils sont utilisés dans les fasciites nécrosantes, ces pansements ne sont pas mis en place initialement mais seulement après plusieurs jours de pansements classiques ce qui permet de s’assurer que l’infection est jugulée et que tout tissu nécrotique a bien été excisé [8,9].

## Conclusion

Les cellulites cervicales sont des affections graves nécessitant un traitement urgent et une prise en charge médicochirurgicale pluridisciplinaire. Le traitement des cellulites cervicales obéit à un principe intangible, celui de traiter une infection en la documentant par des prélèvements bactériologiques et simultanément d’éradiquer sa porte d’entrée, ici très souvent d’origine dentaire. La présentation clinique peut parfois sousestimer l’étendue de l’infection, avec notamment une diffusion au médiastin. Pour préciser au mieux l’extension de l’infection une tomodensitométrie cervicothoracique est nécessaire. Elle permet de guider au mieux la stratégie chirurgicale et l’indication d’une thoracotomie associée à la cervicotomie. Le traitement antibiotique repose sur une association d’antibiotiques, avec en première intention l’administration par voie parentérale de pénicillines A (amoxicilline) avec les 5-nitro-imidazolés. L’évolution en l’absence de traitement se fait vers la médiastinite. Les médiastinites, surtout lorsqu’elles s’étendent en dessous de l’arc aortique, augmentent la mortalité et la durée moyenne de séjour.

## Conflits d’’intérêt

Les auteurs ne déclarent aucun conflit d’intérêt.

## Contribution des auteurs

Issam Serghini a participé à la prise en charge pré, per, et post opératoire du patient et a rédigé l’article. Younés Aissaoui a participé à la prise en charge pré, per, et post opératoire du patient. Youssef Quamouss a participé à la prise en charge post opératoire du patient. Rachid Sedikki a participé à la prise en charge post opératoire du patient. Karim Filali a participé à la prise en charge pré, per, et post opératoire. Mohamed Zoubir a participe à la prise en charge pré, per, et post opératoire du patient et a contribué à la correction de l’article. Mohamed Boughanem a contribué à la correction finale de l’article. Tous les auteurs ont lu et approuvé la version finale du manuscrit.

## Figures and Tables

**Figure 2:**
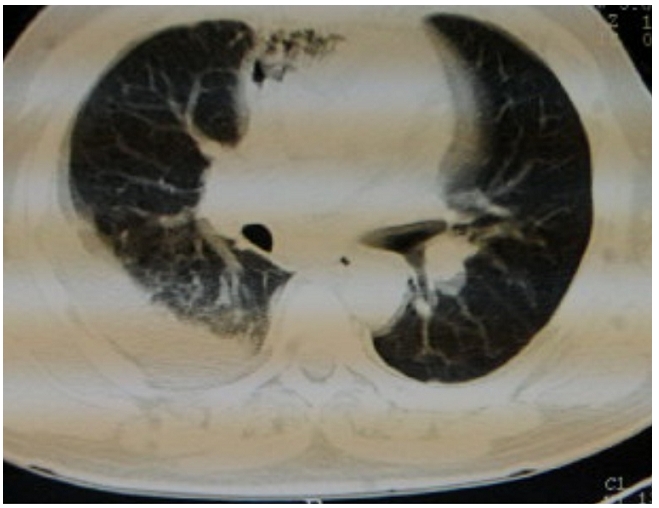
TDM thoracique injectée montrant la médiastinite associé à un épanchement pleural droit chez un patient marocain de 55 ans, diabétiques, avec médiastinite compliquant une cellulite cervicale à porte d’entrée dentaire

## References

[1] Petitpas F, Mateo J, Blancal JP, Mimoz O (2010). Fasciites cervicales nécrosantes. Le Praticien en Anesthésie Réanimation.

[2] Erysipèle et fasciite nécrosante : prise en charge (2001). Conférence de consensus. Ann Dermtol Venereol.

[3] Mathieu D, Neviere R, Teillon C, Chagnon JL, Lebleu N, Wattel F (1995). Cervical necrotizing fasciitis : clinical manifestations and management. Clin Infect Dis.

[4] Mohammedi I, Ceruse P, Duperret S, Vedrinne JM, Boulétreau P (1999). Cervical necrotizing fasciitis: 10 years' experience at a single institution. Intensive Care Med.

[5] Bédos J (2006). Dermohypodermites bactériennes nécrosantes et fasciites nécrosantes : quels antibiotiques et comment?. Annales Francaises d'Anesthésie et de Réanimation.

[6] La Rosa J, Bouvier S, Langeron O (2008). Prise en charge des cellulites maxillo-faciales. Le Praticien en Anesthésie Réanimation.

[7] Rapport HAS (2007). Oxygénothérapie hyperbare. Service évaluation des actes professionnels.

[8] Bronchard R, De Vaumas C, Lasocki S, Jabbour K, Geffroy A, Kermarrec N, Montravers P (2008). Vacuum-assisted closure in the treatment of perineal necrotizing skin and soft tissue infections. Intensive Care Med.

[9] Oczenski W, Waldenberg F, Nehrer G, Kneifel W, Swoboda H, Schwarz S, Fitzgerald RD (2004). Vacuum-assisted closure for the treatment of cervical and mediastinal necrotizing fasciitis. J Cardiothorac Vasc Anesth.

